# Natural Peptide Toxins as an Option for Renewed Treatment of Type 2 Vasopressin Receptor-Related Diseases

**DOI:** 10.3390/biology12040544

**Published:** 2023-04-03

**Authors:** Nicolas Gilles

**Affiliations:** CEA, SIMoS, Département Médicaments et Technologies pour la Santé (DMTS), Université Paris-Saclay, 91191 Gif-sur-Yvette, France; nicolas.gilles@cea.fr; Tel.: +33-6-20840228

**Keywords:** V2R, therapy, natural peptide, animal toxin, hyponatremia, ADPKD

## Abstract

**Simple Summary:**

The type 2 vasopressin receptor is a perfect representative of therapeutic targets that are underexploited due to the lack of safe and effective drugs. This receptor is well-known in terms of physiological roles and tissue expression. Both its activation and blockage are associated with several diseases affecting millions of untreated patients. Despite this, only one agonist and virtually one antagonist are currently available but used with many concerns. It is clear that medicinal chemistry has failed to develop good drugs, leaving millions of patients without treatment. Nature remains the primary supplier of new drugs. A natural resource, animal venoms, has clearly not been sufficiently explored and exploited. These venoms are composed of hundreds of toxins, which have evolved over millions of years to be very often selective and affine to their target, making venoms reservoirs of drug candidates. Animal toxins active on the vasopressin type 2 receptor are reviewed herein, and their potential therapeutic interest is highlighted.

**Abstract:**

The type 2 vasopressin receptor (V2R) is expressed in the kidneys, and it is the keystone of water homeostasis. Under the control of the antidiuretic hormone vasopressin, the V2R ensures vital functions, and any disturbance has dramatic consequences. Despite decades of research to develop drugs capable of activating or blocking V2R function to meet real medical needs, only one agonist and one antagonist are virtually used today. These two drugs cover only a small portion of patients’ needs, leaving millions of patients without treatment. Natural peptide toxins known to act selectively and at low doses on their receptor target could offer new therapeutic options.

## 1. Introduction

The type 2 vasopressin receptor (V2R) is a G-protein-coupled receptor (GPCR) encoded by the *AVPR2* gene located on chromosome Xq28 [1]. It belongs to the vasopressin receptor family, which includes V1a, V1b and oxytocin receptors. V2R is essentially expressed in the kidney and regulates water homeostasis under the control of the antidiuretic hormone vasopressin (AVP [2]). AVP and oxytocin were the first peptide hormones to be characterized structurally and chemically synthesized as biologically active forms in the early 1950s. Vincent du Vigneaud, an American biochemist was the recipient of the 1955 Nobel Prize in Chemistry for AVP synthesis. Seventy years later, the structure of the V2R was solved [3,4,5,6]. V2R is a largely studied and understood receptor in term of functions, signaling pathways and body localization. It is a validated therapeutic target for several diseases such as diabetes insipidus, hyponatremia, heart failure and polycystic kidney diseases. However, unfortunately for the millions of patients who deserve safe and efficient drugs, its pharmacopeia is poor. Indeed, only one agonist and one antagonist are virtually used and, with many concerns, leaving 90% of eligible patients untreated. All these drugs were developed during the end of the last century. The pharmaceutical industry seems to have given up on developing a new generation of drugs with better risk/benefit balances. Consequently, it is time to change the paradigm with respect to the conception of new drugs and to look back to nature, the first source of novel molecules. This review, after a description of the structure and functions of the V2R, cites the most recent findings about animal toxins active on the V2R and their potential applications.

## 2. V2R Structure and Function

The V2R belongs to the AVP/OT receptor subfamily of GPCRs, represented by four receptors: V1aR, V1bR, V2R and OTR. They all show a high degree of sequence identity, with more than 30% of conserved residues among the 370–420 amino acid residues of the human receptor family. In addition, phylogenetic organization based on GPCR sequences or ligand similarities places all four receptors on the same branch, very close to each other [7].

### 2.1. V2R Structures

**Figure 1:**
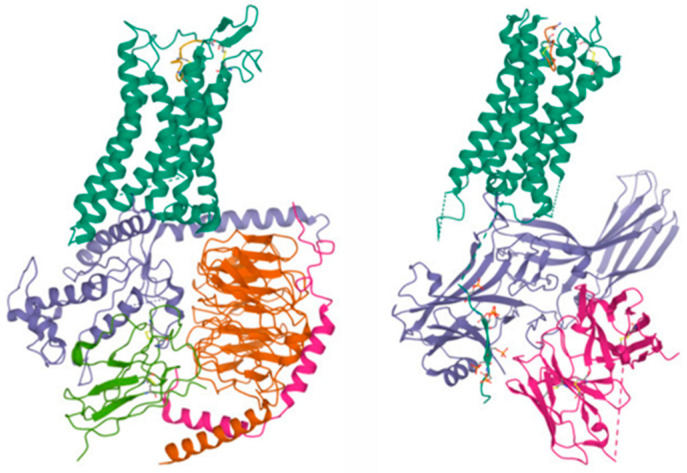
Cryo-EM structures of the V2R complexed with AVP and associated with either the trimeric Gs protein (left, referred to as “T state”, PDB: 7BB7) or β-arrestin2 (right, PDB: 7r0c). Taken from PDB.

Three V2R structures have been solved very recently (Figure 1). The first describes the structure of the AVP–V2R–Gs complex. It contains all residues of AVP (residues 1–9), the GαsRas-like domain, Gβγ subunits, Nb35, scFv16 and V2R residues T31 to L339^8.57^ [8]. The second describes the AVP–V2R–Gs–Nb35 complex with wild-type V2R residues T31 to G345. Only two mutations, N22Q to avoid N-Glycosylation and C358A to limit intermolecular disulfide bridges, have been introduced [5]. The third describes the AVP–V2R–βarr1∆CT–ScFv30 complex [4]. The V2R has a widely open extracellular domain adapted to the cyclic AVP peptide. The orthosteric binding pocket of AVP, which displays affinities between 0.3 and 2.6 nM independent of the vasopressin-sensitive receptors, is composed of all transmembrane helices (TM) and extracellular loops (ECL). The cyclic portion of AVP inserts deep into the core of the transmembrane domain, whereas its C-terminal region is close to the ECLs of the receptor. All nine residues appear to be in contact with the receptor, with polar and hydrophobic contacts. Cys1 makes an H-bond network with Q96^2.61^, K116^3.29^ and Q119^3.32^. Tyr2 forms H bonds with L312^7.40^ and Q174^4.60^. Its hydroxyl part is claimed to be critical to AVP-induced V2R activation. Phe3 is buried in a hydrophobic environment constituted by M120^3.33^, M123^3.36^, Y205^5.38^, V206^5.39^, I209^5.42^, F287^6.51^, F288^6.52^ and Q291^6.55^. Gln4 makes polar contacts with R202^5.35^ and Q291^6.55^. Asn5 faces A194^ECL2^, and Arg8 forms salt bridges with D33^1.28^ and E40^1.35^. Finally, the C-terminal amidated extremity makes contact with residues from the top of TM1 and TM2 and with ECL1 and ECL2: E40^1.35^, K100^2.65^, D103^ECL1^, R104^ECL1^ and W193^ECL2^. The basic Lys100 is specific for the human V2R, and rats, mice and pigs have an aspartic acid residue at this position.

The V2R, when coupled with Gs, induces cAMP production. Interestingly, there is a direct contact with the side chain of R137^3.50^, which is part of the ionic lock motif and the free carboxylic acid function of the Gs C terminus. This was never observed before between a GPCR and a G protein of any family (Gs, Gi, Go or Gq). The Gs protein, as well as the β-arrestin1, insert into a core cavity formed by the outward movement of the TM6. The α-subunit of the Gs Cter protein (helix h5) and the tip of β-arrestin1 overlap in the same area. This interaction forces β-arrestin1 into an atypical position that has not been previously described. All of this structural information provides evidence to begin to better understand the function of the V2R.

### 2.2. V2R Natural Mutations

Over 260 mutations have been reported to date on hV2R (the Human Gene Mutation Database at the Institute of Medical Genetics, Cardiff, UK) and recently reviewed [9]. These mutations are associated with either loss or gain of function associated with human diseases. Recent V2R structures are helping to understand the physiological role of their mutations and, hopefully, to design drugs that can counteract their effects. For example, in most class A GPCRs, D136^3.49^ and R137^3.50^ interact with each other through an ion lock and are key points in the balance between active and inactive states. In the V2R, R137C or R1373L missense mutations induce constitutive activity toward Gs coupling. This activity, inducing water retention and hyponatremia, can be explained from a structural point of view.

### 2.3. V2R Expression and Functions

The V2R, like many GPCRs, induces pleiotropic actions by G-protein-dependent and G-protein-independent mechanisms. Numerous and exciting works are underway to evaluate, through artificial cell constructs, how a receptor could interact with a maximum number of effectors. However, the physiological relevance of each of these interactions detected in cellular assays has yet to be demonstrated in tissues. Beyond the three classical V2R signaling pathways, which are phosphorylation of Gs, β-arrestin-1 and MAP kinase [10], other G proteins seem to interact with the V2R. According to Bouviers’ strategy, the V2R interacts with Gq, Gq11, G13, G14 and G15 [11]. According to Inoue’s strategy, the V2R interacts with only Gq and Gq11 [12].

Human V2R expression in nephron has been established by mRNA localization. V2R mRNA was identified in the thick ascending limb and connecting tubules and at a lower level in the distal convoluted tubule [13]. In the cortical and outer medullary collecting duct, AVP stimulates sodium reabsorption through the epithelial sodium channel ENaC. In the terminal inner medullary collecting duct, AVP enhances urea permeability by activating urea transporters UT-A1 and UT-A3 [14,15], and in the thick ascending limb, AVP stimulates the Na-K-2Cl cotransporter NKCC2 and thus promotes sodium reabsorption. The V2R/Gαs voice induces intracellular production of cAMP, which activates protein kinase A to phosphorylate aquaporin 2. Once aquaporin 2 (AQP2) is phosphorylated, vesicles fuse with the apical membrane via a mechanism of calcium-dependent intracellular exocytosis. This mechanism occurs throughout the collecting duct. Luminal fluid passes through aquaporin 2 at the apical membrane and reaches the main cell before passing into the blood via aquaporins 3 and 4 at the basolateral membrane [16].

The V2R is also expressed outside the kidney, such as in the inner ear, where it can regulate the hydraulic pressure of the endolymphatic system [17]. An elevated expression level of the V2R [18,19] and AQP2 at the mRNA and protein levels [18] has been established in the endolymphatic system of Menière’s disease patients [17]. Interestingly, in vestibular EC5v cells, AVP receptors have been clearly identified, but no cAMP was produced once stimulated [20]. In bone, one publication suggests that the V2R contributes to homeostasis [21]. The V2R is also localized in the vascular endothelium. V2R activation induces endothelium-dependent NO release that can vasodilate the smooth muscle cells [22]. V2R mRNA was identified in whole-lung and in cultured-lung microvascular endothelial cells [23], as well as in fetal lung, where AVP contributes to the reabsorption of alveolar fluid in the hours following birth [24]. Finally, V2R mRNA was also described in several cancer cells, such as breast cancers [25,26,27], colorectal cancer [28], non-small cell lung cancer [29], prostate cancer [30] and renal carcinoma [31]. Only one publication has certified the presence of active V2R at the protein level expressed at the surface of renal cancer cell lines thanks to a fluorescent snake toxin [32].

The effects of V2R agonists and antagonists on the viability of several V2R-expressing cell lines have been tested, as well as, more interestingly, on in vivo models. Both agonists and antagonists have beneficial effects, depending on the nature of the cancer. The antitumor effects of dDAVP in syngeneic mice bearing F3II, a breast cancer model, was demonstrated [26,33]. [V4Q5]dDAVP, an analog of dDAVP, inhibited the proliferation of hepatic and pulmonary metastases in syngeneic mice surgically implanted intrasplenically with CT-26 cells, a murine colorectal cancer cell line. On the other hand, in renal carcinoma cancer, it is the antagonist OPC31260 that inhibits tumor growth in xenograft mice inoculated with Caki-cells [31].

### 2.4. AVP Secretion

Circulating AVP concentrations are generally very low (in the pM range). Vasopressin is primarily secreted by AVP neurons in the supraoptic and paraventricular nucleus of the hypothalamus. AVP neurons receive signals from the lamina terminalis, which detects changes in osmolality and modulates thirst and water retention [34]. Both eating and drinking have profound effects on osmolality in our body. The main stimulus for its release is dehydration, resulting in an increase in plasma osmolality, with sodium concentration having a larger influence on AVP secretion [35,36]. It has been proposed recently that AVP neurons respond to food and water intake by a rapid adaptation of their activity [37]. Indeed, AVP levels drop within minutes of water consumption, even before changes in blood osmolality occur. These results suggest that AVP neurons possess the ability to anticipate future osmotic changes that will occur following food and water intake. This hypothesis is reinforced by another recent publication [38]. It is suggested that excitatory and inhibitory neurons in the lamina terminalis help to anticipate future osmotic changes by reducing AVP activity in response to water intake. Thirst has long been considered a negative homeostatic response to increases in blood solute concentration or decreases in blood volume. However, emerging evidence suggests that thirst plays a clear role as an anticipatory feedforward adaptive response that precedes physiological challenges. Anticipatory signals are also important mediators of satiety, inhibiting thirst long before the physiological state is restored by fluid intake [39,40].

AVP also seems to be produced outside the central nervous system. A recent and interesting result suggests that epithelial cells of the kidney collecting duct also produce AVP in response to water deprivation [41]. The heart also seems to be an organ of AVP production, unless under certain pathophysiological conditions. Indeed, a significant increase in copeptin, a valid marker of vasopressin [42] in patients suffering from acute myocardial infarction, has been established [43]. Finally, AVP is also secreted by several tumors. This ectopic production has been demonstrated in lung cancers [44]. Under these pathophysiological conditions, the excess of circulating AVP induces hyponatremia, which is deleterious for patient conditions.

## 3. V2R, an Unexploited Target, Links to Unmet Therapeutic Needs

GPCRs are of major therapeutic interest and are targeted by 30% of currently available FDA-approved drugs. The V2R, due to its central role in water homeostasis, is associated with a wide range of diseases. Despite its importance, its pharmacopeia is poor and does not meet all needs.

Only one agonist drug is on the market, which is used for central diabetes insipidus and enuresis: dDAVP (Minirin^®^, 1-desamino-8-d-arginine vasopressin), which was published in the late 1960s [45] and later licensed to Ferring Pharmaceutical (St-Prex, Switzerland). dDAVP is a V2R-selective agonist in rat with affinities for V1aR, V1bR and V2R of 100 nM, 9.29 nM and 0.3 nM, respectively. On human receptors, dDAVP is not selective, with affinities of 62.4 nM, 5.8 nM and 23.3 nM for V1aR, V1bR and V2R, respectively [46]. The main advantage of dDAVP lies in its pharmacokinetics. In humans, AVP has a plasmatic half-life of between 10 and 20 min [47], while dDAVP has a half-life of between 2.7 and 4.6 h [48].

Three antagonists of the V2R are on the market. Conivaptan (Cumberland Pharmaceuticals, Nashville, TN, USA) is accepted only in the USA by IV administration. Mozavaptan (Otsuka Pharmaceuticals, Tokyo, Japan) is only accepted in Japan. Tolvaptan (Otsuka Pharmaceuticals, Tokyo, Japan) is accepted in many countries for hyponatremia and ADPKD and is virtually the only antagonist used.

Hyponatremia is defined by plasma sodium concentrations below 135 mmol/L. This syndrome is associated with an increase in AVP secretion that can be related, for example, to central neuronal disorders, the presence of certain tumors, or heart and/or kidney failure. Hyponatremia is a serious problem that should be taken into account as it is associated with increased comorbidity. Certain medications, including loop diuretics and thiazide diuretics, could induce hyponatremia [49]. The most efficient way to restore physiological natremia is to remove water without loss of electrolytes. Blocking the V2R induces this effect, called a aquaretic effect. Pharmaceutical industries, such as Astellas, Sanofi, Otsuka Pharma and Cardiokine, in the 1980s and 1990s, developed so-called vaptans, mostly from benzazepine structures [50]. Tolvaptan is the most used vaptan but is associated with many concerns due to its hepatotoxicity [51].

V2R blockade was also validated earlier this century for ADPKD [52]. PKDs are genetic diseases with autosomal dominant and recessive forms affecting 1 in 1000 and 1 in 20,000 people, respectively. While many therapeutic strategies have been tested against ADPKD, only V2R blockade has been proven effective in humans [53]. The two main side effects associated with tolvaptan reported by physicians are nocturia and liver toxicity. Tolvaptan is mostly used for rapidly progressive ADPKD patients, and disparities in it use across continents and countries are significant. Tolvaptan is widely prescribed in Japan, the country of Otsuka Pharma (Tokyo, Japan). It is well accepted in the United States but much less so in Europe. It is estimated that fewer than 10% of patients eligible for treatment take tolvaptan, leaving 90% of patients untreated.

## 4. Natural Peptide Toxins Targeting V2R

Peptide toxins in venoms have undergone a long evolutionary process, allowing for host defense or prey capture and making them highly selective and potent for their target. This has led to the emergence of a wide panel of toxins from a large variety of species with diverse structures and multiple associated biological functions. In this way, animal toxins constitute an inexhaustible reservoir of drug molecules with interesting pharmacological properties.

Toxins present a great diversity in terms of size (from 10 to 80 amino acids), with up to six disulfide bridges, and belong to a wide panel of structural scaffolds. It is estimated that the 200,000 species of venomous animals on Earth could produce about 40 million toxins, of which only 7000 have been characterized [54]. Furthermore, the biological effects and molecular targets of many toxins present in venoms are still unknown, which explains why venom screening to identify new ligand–receptor pairs has gained momentum.

Due to a specific evolutionary process that occurs independently in many animal lineages, the great pharmacological qualities of toxins explain their major impact on the hemostatic, cardiovascular or peripheral nervous systems of prey. Toxins are largely known for their activities in ionic channels and the cardiovascular system, which have been reviewed for conus [55], snake [56], scorpion [57], spider [58], centipede [59] and sea anemone [60]. An increasing number of animal toxins have also been described to be active in GPCRs, with an interesting classification: agonist-mimicking toxins and non-related agonist toxins, as recently reviewed in [61]. Here, both families of toxins active on the V2R are detailed.

### 4.1. AVP-like Toxins

The vasopressin–oxytocin family of peptides is of very ancient lineage and found in organisms as diverse as hydra and man. AVP-like toxins have been found only in cone snails and named conopressin (Table 1). A total of 15 conopressins are classified in the ConoServer database (https://www.conoserver.org/ accessed on 8 March 2023), which have been described since 1987 [62]. Most pharmacologically characterized conopressins show activities on V1a/b receptors as agonists or antagonists. Conopressins strictly conserve the AVP cysteine residues in positions 1 and 5 to keep the cyclic structure. The hydroxyl group of the AVP TYR2 is rarely conserved in conopressin. The AVP PHE3 is never conserved in conopressins but is occupied by hydrophobic residues, which are probably compatible with the hydrophobic pocket of the V2R binding site. The Gln4 of the AVP has been extensively studied, and any changes induce loss of affinity for the V2R [63]. This position, which has polar interactions with the V2R, is conserved in only two conopressins, the others having a small residue (GLY or THR) or a basic ARG. Finally, ARG 7, one of the most important positions for AVP function, is conserved (with LYS) in the six closest conopressin sequences compared to AVP. For other conopressins, this position is mainly occupied by a negatively charged residue that is probably incompatible with V2R interaction.

Among the seven conopressins tested on hV2R, only the G conopressin showed modest agonist activity [64]. Conopressin G lacks the hydroxyl group of the TYR 2, with a PHE instead, which suggests that this hydroxyl group is not as essential for V2R activation as described in [3]. This assumption is supported by the APC2-AVP, in which the TYR2 is replaced by an amino cyclopentane group, which is three times more potent than AVP to induce antidiuretic action in rodents [63]. Finally, the Gln4 of AVP is an ARG in conopressins G, which is deleterious due to its affinity for the V2R.

### 4.2. The Non-Related AVP-like Toxins

Until now, 11 “non-related AVP-like” toxins have been known to bind the V2R. They are all snake Kunitz-type peptides. The first Kunitz peptide, the bovine pancreatic trypsin inhibitor was described in 1936 and inhibits serine proteases [67]. Dendrotoxins from mamba snake venoms were described to block potassium channels [67,68]. The first identified V2R toxin called mambaquaretin-1 (MQ1) was found in *Dendroaspis angusticeps* venom [10]. In a second step, the three other mamba snake venoms—those of *Dendroaspis jamesoni*, *Dendroaspis polylepis* and *Dendroaspis viridis*—were screened, and eight other mambaquaretins were identified [69]. The representativeness of mambaquaretins in these venoms ranges from 0.15 to 0.8%, so they are ultra-minority toxins. A phylogenetic analysis of these nine mambaquaretins showed that they represent a distinct and novel subgroup within the Kunitz-type peptides of snakes. This analysis also revealed the presence of a group of two Asian cobra toxins close to the mambaquaretin toxins. P19859 and Q5ZPJ7 are weakly characterized as serine protease inhibitors [69]. They are also V2R antagonists, demonstrating that this activity is not limited to the mamba species. The next phylogenetic group of toxins is composed of dendrotoxins; interestingly, none of them are active on the V2R. The mambaquaretins are a new group of Kunitz-like peptides associated with a new function. Among these nine mambaquaretins, MQ1 is the most studied.

## 5. From a Natural Toxin to a Drug Candidate?

MQ1 binds V2R with an affinity of between 2.5 and 5 nM. Tested on Kv1.1 and trypsin, the original targets of Kunitz’s peptides, MQ1 showed activities only at 25 µM. In addition, MQ1 is not active on 155 other GPCRs, 15 ion channels and 45 proteases, making this peptide the most selective V2R antagonist ever found. When tested on all three V2R signaling pathways, Gs, β-arrestin-1 and MAP kinase phosphorylation, MQ1 showed a pure competitive antagonist effect [10].

### 5.1. In Vivo Activity of MQ1

In vivo blockade of the V2R is associated with an increase in diuresis. In rats and mice, administration of MQ1 by IP or SC injection routes induces a dose-dependent increase in aquaresis associated with a decrease in urinary osmolality, which is characteristic of an aquaretic effect [10,32]. As previously mentioned, V2R blockade is a validated therapeutic strategy in humans for hyponatremia and polycystic kidney disease. MQ1 was highly effective in restoring natremia in a rat model of hyponatremia at doses as low as 20 µg/kg [32]. The CD1-*pcy*/*pcy* mouse strain suffers from type 3 nephronophthisis, similar in many aspects to ADPKD [70]. Mice treated daily with 12 µg MQ1 i.p. for 99 days developed 30% fewer cysts with smaller sizes than those in the control group [10].

### 5.2. MQ1/V2R Mode of Interaction and Engineering

**Figure 2:**
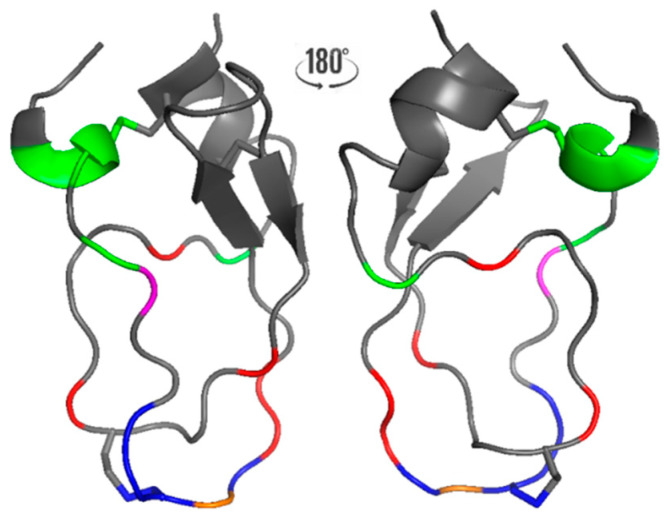
X-ray structure of the variant MQ1 NG15/16KA (PDB 5m4v). Colors identify pharmacophores: green for the dendrotoxin, blue for trypsin inhibitor and red for MQ1. Overlapping pharmacophores between dendrotoxin and MQ1 are indicated in magenta, and those between the trypsin inhibitor and MQ1 are indicated in orange.

BPTI-type peptides interact with serine proteases through loop 1 via four residues, two of which are essential: the K/R15 and G/A16 dyad [71,72]. Dendrotoxins block the Kv1.1 potassium channel, predominantly with its lysine and an aliphatic residue positioned in the N-terminal extremity of the peptides [73,74]. These two pharmacophores are diametrically opposed in the Kunitz structure. Neither the N and C terminus of MQ1 nor its structured part are involved in V2R binding. MQ1 uses a surface defined by its two main loops that spills over the pharmacophores of BPTI and dendrotoxin (Figure 2).

The N-terminal extremity of the MQ1 was exploited to generate imaging tools. A fluorescent toxin (coupled to cy3 or Cy5.5) identified V2R expression in several cancer cell lines [32] for the first time at the protein level. At the same position, a deferoxamine (DFO) was coupled to the MQ1. DFO chelates ^89^Zr, a positron emission tomography (PET) emitter. Injected in mice, the biodistribution showed that ^89^Zr-MQ1 can be detected in organs of elimination such as the liver and the gallbladder but in a large majority in the kidney. In fact, apart from the kidney, no other organs were imaged by ^89^Zr-MQ1, showing its in vivo selectivity for this organ.

## 6. Discussion, Conclusions and Future Directions

Traditional medicine has always turned to nature as a source of care, and even today, many active components are found in the animal and plant kingdoms. Not surprisingly, they represent nearly 40% of therapeutic targets, with 460 FDA-approved drugs in 2020 and approximately 60 in clinical trials [75].

Animal venoms are seen as a dangerous cocktail of poisons. They can also be considered as a reservoir of optimized peptides with exquisite pharmacological properties. The conopressin toxins described today do not yet show pharmacological properties suitable for V2R therapy, but hope is permitted. For example, exenatide from the giant Gila monster *Heloderma suspectum* is a GLP-1-mimicking toxin with improved metabolic stability for type 2 diabetes with billions of dollars in sales [76]. Another example of nature’s beauty in developing effective molecules is Consomatin Ro1 from conus rolani. Consomatin Ro1 is an evolutionarily optimized stable somatostatin analog [77]. Somatostatin hormone is a 14-residue cyclic peptide that acts on the 5 somatostatin receptors. It has been designed in Octroetide (Sandostatin) as a drug for diseases such as hypersecretory tumors, diabetes, cancer, pain and inflammation [78]. Octreotide has improved metabolic stability due to the reduction in its loop size from 10 to 4 residues and the introduction of a d-TRP. Astonishingly, Consomatin Ro1 already exploited these combinations of a four-residue loop and a d-TRP positioned at the same location as octreotide. These two examples of exenatide and Consomatin Ro1 illustrate how nature, with its millions of years of evolution, provides optimized molecules ready to be used for human health. Although no conopressin with the right properties for V2R has yet been identified, it is likely that further exploration of the 900+ conus classified today may provide effective agonists.

MQ1, which is derived from the African green mamba snake, combines all the in vitro and in vivo properties to become a drug candidate. MQ1 is the V2R antagonist with the largest therapeutic window in rodents, which is promising for the development of safer drugs than vaptans. The classical strategy of developing small molecules following Lipinski’s five rules failed for V2R [79]. The paradigm shift induced by the exploration of an unexpected source of V2R peptides, namely venoms, has been successful and may stimulate the exploration of venoms directed to any therapeutic targets of interest. Indeed, several GPCR toxins with original activities and promising applications have been described recently. These toxins act on the M2 muscarinic receptor implied in arterial contraction [80], on the melanocortin type 4 receptor implied in energy homeostasis [81,82] and on angiotensin receptors implied in cardiovascular homeostasis [83].

The diversity of toxin activities combined with high selectivity and affinity are associated with novel or unexpected pharmacological properties with potential applications in drug discovery and development.

## Figures and Tables

**Table 1:** Conopressin sequences and functions on V2R.

Peptide					V2R	
Name	Sequence	id%		Affinity		Function
AVP	CYFQNCPRG *	100				
Ba1	CY**IT**NC**O**RG *	70		ND		ND
Ba3	C**FIR**NCPRG *	70		ND		ND
S	C**IIR**NCPRG *	70		>10 µM (1)		
T	CY**I**QNC**L**R**V** *	70		>10 µM (1)		
**G**	C**FIR**NCPKG *	70		299.2 nM (2)		agonist (2)
M	C**FIR**NCP**E**G *	60		ND		ND
Vil	C**LI**Q**D**CP**(Gla)**G	50		ND		ND (3)
M1	C**FPG**NCP**DS** *	50		>100 µM (2)		
M2	C**FLG**NCP**DS** *	50		>100 µM (2)		
Ba2	C**FLG**NC**LND** *	40		ND		ND
Ml1	C**FPG**NCP**DS**	40		>100 µM (2)		
Cn	C**YIRD**CP**E**- *	40		ND		ND
Tx	C**FIR**NCP**O**	40		ND		ND
Ml2	C**FLG**NCP**DS**	40		>100 µM (2)		

(1): [65]. (2): [64] (3): [66]; ND: not determined. id%, sequence identity compared to AVP. *: C-terminal amidated.

## Data Availability

Not applicable.
